# Impact of COVID-19 pandemic on African indigenous vegetables value chain in Kenya

**DOI:** 10.1186/s40066-021-00328-3

**Published:** 2021-12-06

**Authors:** Maurice Juma Ogada, Ochieng’ Justus, Maina Paul, Sikei Geophrey Omondi, Adero Nashon Juma, Evans Taracha, Hassan Ahmed

**Affiliations:** 1 Taita Taveta University, Voi, Kenya; 2 Bayesian Consulting Group Limited, Nairobi, Kenya; 3 Independent Scholar, Nairobi, Kenya; 4 Natural Products Industry (NPI) Initiative, National Museums of Kenya (NMK), Nairobi, Kenya

**Keywords:** COVID-19, African indigenous vegetables, Kenya

## Abstract

**Background:**

African indigenous vegetables are important for food security and nutrition, and income of the poor farm households. In the era of COVID-19, they are critical for boosting people’s immunity. Unfortunately, both production of and trade in these vegetables is likely to be severely affected by the pandemic.

**Methods:**

This study examined potential effects of COVID-19 pandemic on production and trade of African indigenous vegetables using a cross-sectional survey of 244 farmers and 246 traders from different regions in Kenya.

**Results:**

COVID-19 has a negative impact on production and trading of AIVs in Kenya. Findings indicate that 75% of the farmers are experiencing declining production due to reduced access to input, farm labour and output market. Secondly, about 98% of the traders have recorded a drop in sales volumes due to containment measures implemented by the government and personal safety precautions. In particular, farmers’ production and traders’ sales volumes declined by 39 and 65%, respectively, during the first phase of the pandemic.

**Conclusion:**

The findings indicate that the sub-sector requires targeted interventions which may include input support, careful reopening and control of the open-air markets, reduced taxation and facilitated access to urban markets.

## Background

Africa indigenous vegetables (AIVs) are nutritious (rich source in minerals and vitamins) and are well adapted to local conditions [1]. Consumption of AIVs such as African nightshade (*Solanum nigrum *L*.*), amaranth (*Amaranthus hybridus *L.), spider plant (*Cleome gynandra *L*.*), jute mallow (*Corchorus olitorius *L*.*), Cowpea leaves (*Vigna unguiculate *L*. Walp*), Crotalaria and Okra ensures that staple-based diets are balanced and provide both food and nutrition security and income. These vegetables have high levels of minerals, especially calcium, iron and phosphorus, vitamins A and C and proteins [2]. Thus, they are important, especially to the vulnerable groups such as pregnant and nursing mothers and children under 5 years old. For example, amaranth has 30 times more vitamin A per 100 g than common cabbage [3]. AIVs can be grown intensively on small plots of land and are mostly grown by women, mainly for home consumption with surplus sold at the local markets. A recent study conducted by the National Museums of Kenya showed that AIVs in Kenya are grown on small plots of land (less than 0.1 Ha) but producers still achieve positive gross margins [4]. AIVs have been part of diets in Kenya for generations as part of the diverse culture of the Kenyan people but their prominence was relegated to being ‘poor man’s diet’ with introduction of ‘exotic’ food crops during the colonial period. However, their importance has re-emerged, and their demand has been rising due to the promotional efforts by the government and other stakeholders backed by scientific studies showing their superior nutritional properties and changing trends on healthy lifestyles [1, 5, 6].

AIV sub-sector is very delicate, relying on family labour, rainfall and highly volatile market prices. This means that any external shock such as the COVID-19 pandemic is likely to have a significant impact. Measures to contain COVID-19 have significant effects on food value chains especially on perishable horticultural products like AIVs which are vulnerable because of their seasonal labour requirements and high perishability [7]. A study by [7] in Ethiopia showed that COVID 19 impacted vegetable value chains negatively by reducing traded and consumed volumes, producer prices and increased farm losses and reducing farm labour. Appropriate interventions, that are evidence-based, are required to aid recovery of the sector and enable it to become resilient to future shocks. Similarly, a study by [8] in India showed that a majority of vegetable farmers reported negative impacts on production, sales, prices and incomes.

This study was undertaken to assess the impact of COVID-19 and the accompanying government containment and control policies on the production and trade of AIVs in major production and consumption hubs in Kenya. Generally, there is paucity of studies on impact of COVID-19 on agriculture in the developing countries. More important, no previous study has focused on AIVs whose importance for nutrition is rising in Kenya and the region. In recent years, disease and disaster governance have had increased global public policy concern. Novel coronavirus disease (COVID-19), is a classic example of a highly infectious disease that has spread worldwide mainly through community transmission as confirmed by the World Health Organization (WHO). In Kenya, the first COVID-19 case was recorded on March 13, 2020. The country’s recovery rate of 66% is far below Africa’s average of 85% [9]. To control the spread, the government took several measures as summarized in Table 1.

**Table 1:** COVID-19 containment measures in Kenya

Direct containment measures	Other measures
Closure of learning institutions	Short mobile phone messages to remind people of COVID-19 and public health measures
Implementation of night curfews (7 PM–5 AM later revised to 9 PM–4 AM)	Reduction of value added tax rate by 2%
Quarantine and isolation	Reduction of income tax rate by 5%
Cessation of movements across specific Counties but was later lifted in July	Reduction of turnover tax rate by 2%
Closure of bars, restaurants and hotels	Reduction of the resident corporate tax rate by 5%
Closure of open-air markets	Incentives to use mobile money services and e-banking by reducing cost of money transfer services
Termination of international and local air travel	Income support to vulnerable groups
Public health measures: hand washing, use of sanitizers, thermos guns, ban on public gatherings, social distancing, use of face masks and/or shields	Daily update on COVID-19 statistics
Targeted mass testing	
Requirement for civil servant over 58 years and above to work from home	
Encouragement for all workers who can to work from home	

Source: [10, 11]

Containment measures, though successful in taming the spread of COVID-19, have serious socio-economic consequences on agri-food system [7, 8, 12]. Because of the containment measures implemented by governments across the world, COVID-19 pandemic has disrupted almost every sector of the economy, agriculture sector value chains included [13]. These measures have led to disruption in flow of inputs and output to markets. In Kenya, where about 25% of the population is food poor [14], food insecurity is likely to worsen due to reduced availability of and access to food. To understand some of these effects, this study focused on the African indigenous vegetables (AIVs) value chain in Kenya.

## Review of past pandemics and their effects on agricultural value chains

Global pandemics are not new to the world; past pandemics include HIV/AIDS, influenza, Ebola, acute respiratory syndrome (SARS), Middle East respiratory syndrome (MERS) and the 1994 plague outbreak in India [15]. Whenever they occur, pandemics have a disruptive effect on human lives and livelihoods, especially on rural populations which are heavily dependent on primary sectors of the economy (often with low productivity and low wages) [16, 17]. The extent of their impact, however, depends on the resilience of individuals and communities which is shaped by their access to various forms of livelihood capital [18, 19]. Overall, pandemics tend to weaken household assets, whether human (knowledge and ability to work), physical (technology), financial (access to credit) or social (networks and support systems), which are critical for survival [19]. In agriculture, pandemics have both supply side and demand side effects.

In developing countries, crop agriculture is potentially vulnerable to shocks arising from pandemics because of its high dependence on manual labour. For example, crop production in developing countries is labour-intensive, and during pandemics, productive labour gets eroded through illness and death [20, 21]. Furthermore, disease containment measures such social distancing and self-isolation, which typify pandemics, curtail labour mobility, leading to labour scarcity, reduced productivity and higher wages. During Ebola outbreak in West Africa, farms suffered agricultural labour shortages for planting and harvesting as communities stayed away from agricultural fields, resulting in reduced yields and production [22, 23]. In Zimbabwe, smallholder farm outputs dropped by about 50% due to labour shortages associated with the emergence of HIV/AIDS which rendered productive lands idle [24]. Containment measures further results into disruptions to food supply chains impeding the proper functioning agricultural markets for both inputs and outputs [18, 25, 26]. The inability of producers and traders to link agricultural produce to markets result in lost household income, while producers are forced to maintain production activities at higher costs, as inputs become economically and physically inaccessible, affecting yields [27].

Large scale agricultural transformation is dependent on intensification associated with investment in improved agricultural technologies and innovation [28, 29]. Smallholder farms rely on remittances and off-farm income as the primary means of financing the agricultural intensification [30]. Notably, pandemics lead to losses of both farm and off-farm income [31]. The threat is greater among the asset-poor and vulnerable households because these income losses further strain their ability to accumulate productive assets [32]. This is compounded by the increased difficulty in securing agricultural credit, a critical input in enhancing agricultural productivity [25, 33]. Inability to secure credit emanates from either lack of collateral or the economic gloom which makes financial institutions hesitant to lend, especially to sectors considered more vulnerable to the pandemics.

The spread of COVID-19 pandemic has raised a lot of concern on a global, regional, and local scale. Studies have reported disruptive effects of the pandemic and measures taken to contain it on agricultural value chains [7, 34, 35] with farmers and poor rural and urban households worst hit by these effects [7]. Agricultural value chains have been grossly impacted through both, up- and down-stream blockages. In Kenya, onset of the pandemic coincided with the start of the long rains, the peak season for labour-intensive staple food and vegetable production. The lockdown and movement restrictions resulted in farm labour shortages, disruption to access to agricultural inputs, extension, and advisory services, resulting in a decline in yield levels [36].

## Method

### Conceptual framework

The analytical approach employed in this study is borrowed from the Sustainable livelihoods framework (SLF) [37, 38]. SLF is used to understand differential capabilities of rural farm households to cope with stresses or shocks [39] and their ability to achieve sustainable livelihoods [19]. One of the critical aspects of this study is that in addition to producers’ analysis, it also analyses how the traders’ incomes from vegetables have been affected by the pandemic. Using SLF, our study provides policy insight with the objective to reduce the impact of future pandemics on livelihoods. SLF has been used by several studies to explore the livelihood effects of other pandemics such as Ebola virus disease (EVD) [19] and HIV/AIDS [32].

The COVID-19 pandemic directly affects the rural labour force through sickness or death, temporarily or permanently removing casual agricultural labourers which disrupts sourcing for inputs, planting and harvesting and raising farm wages. Besides, farm labour force participation could fall indirectly due to measures to contain the virus, such as travel restrictions or bans, quarantines and efforts to avoid crowds. Limited availability of labour and yield enhancing inputs (fertilizers, improved seeds, crop chemicals, etc.) needed for production would reduce area devoted to AIVs production and subsequently lower yields which eventually reduce the ability of producers to have a sustainable livelihood.

The measures to reduce the spread of the virus and fear of infections directly affect the marketing of AIVs in local, regional and international markets, associated value addition activities, and financial markets. The financial markets are affected through limited credit available for agricultural investments, as loans are not approved by the lending institution, while the ability of the farmers and agro-based small and medium enterprises (SMEs) to repay the existing loans diminishes. This not only affect the income of the AIV value chain players but also the availability of the nutrient-rich AIV products needed to boost immunity against COVID-19.

### Data sources

The study is based on a survey of 244 farmers and 246 traders, carried out as a rapid follow-up of 418 farmers and 446 traders previously interviewed in 2019 in six Counties; Kiambu, Nairobi, Kirinyaga, Kisumu, Migori, Vihiga in Kenya[4] (Table 2). These counties are major production and consumption hubs of AIVs [6, 40]. In the follow-up study, farmer responses were very poor in Migori while trader responses were very poor in Kirinyaga. In each case, the response was about 5% which would not yield meaningful comparison with baseline. Thus, Migori was excluded from the sample of farmer survey while Kirinyaga was excluded from the sample of trader survey. Key informant interviews (KII) were also conducted with varied experts on the AIV value chain including county government officials, lead researchers and market leaders.

**Table 2:** Distribution of the sample size in the study sites and response rate

County	Farmers		Traders	
	2019	Follow-up survey, 2020	2019	Follow-up survey, 2020
Kiambu	100	72	67	45
Kirinyaga	109	45	50	–
Kisumu	51	41	46	23
Migori	51	–	52	30
Vihiga	107	86	49	29
Nairobi	–	–	182	119
Total (*N*)	418	244	446	246
Response rate (%)		66		62

We linked the follow-up survey with background information of the farmers and trader’s data collected through the support of Natural Products Industry Initiatives (NPI) and National Museums of Kenya (NMK) in 2019. While phone interviews are known to record low response rates, we note that 66% of farmers and 62% of traders previously covered in face-to-face interviews in 2019 responded to our interviews. This response rate can be described as above average (see [41]).

**Fig. 1:**
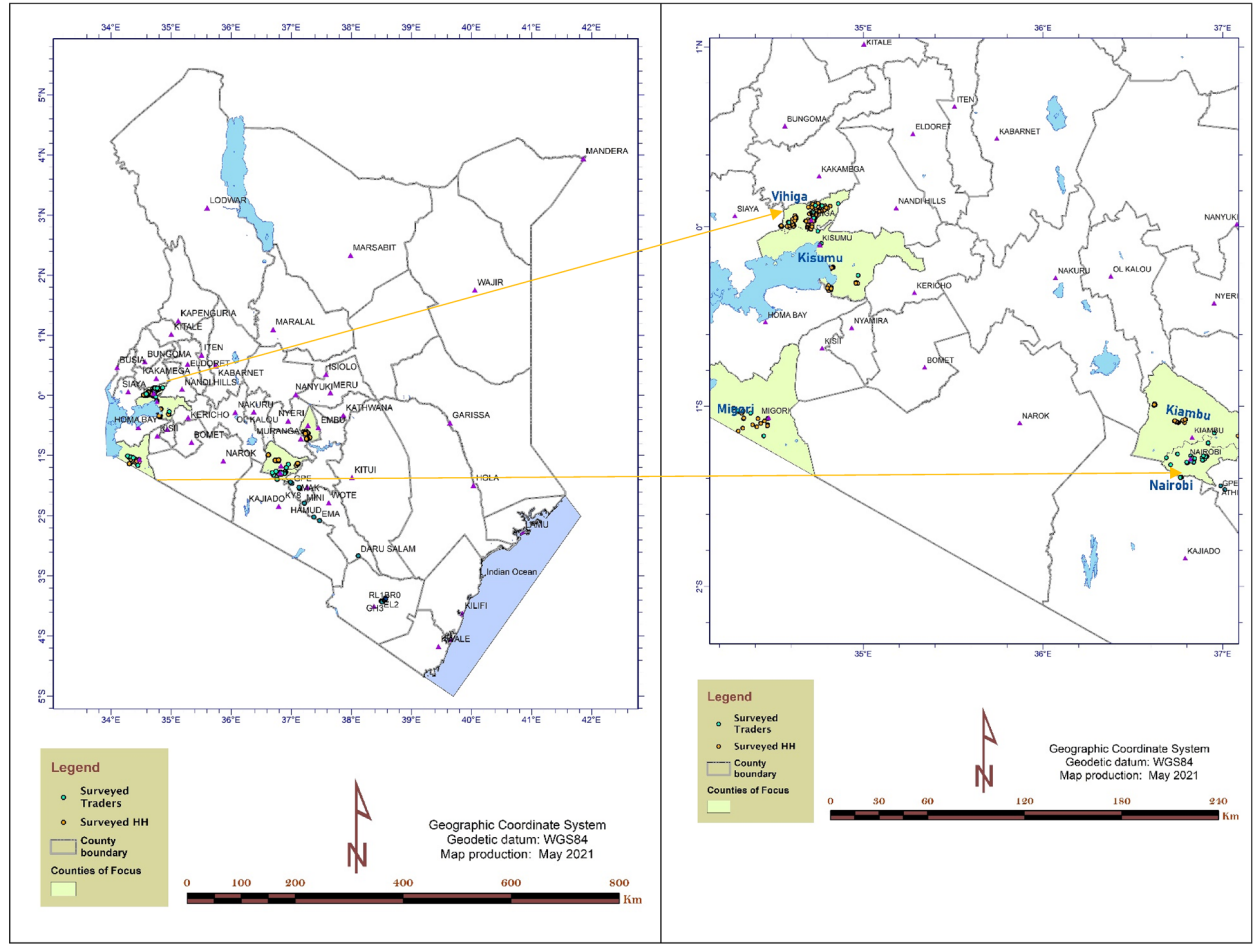
The surveyed households and traders across Kenya

The spatial spread of the surveyed farm households and the traders is shown in Fig. 1. Kisumu, Migori and Vihiga are in Western Kenya, Kiambu is in Central Kenya, Kirinyaga is in Eastern Kenya while Nairobi is in the Nairobi Metropolitan Region.

### Data collection and analysis

Data collection was conducted remotely through phone interviews due to the pandemic lockdown conditions in Kenya. Three enumerators were trained and deployed for data collection. Farmer and trader questionnaires were administered through computer assisted personal interviews (CAPI) for speed, accuracy and ease of monitoring.

At the time of interview, COVID-19 was a serious topic of discussion in both social and mainstream media across the country and the respondents easily accepted to answer the questions and expressed their perceived impacts of the pandemic on their farming and trading business operations. Farmers were asked about the resumption of AIV production activities while the traders were asked about the trading activities such as if the current cash flow would sustain their survival. Both farmers and traders were asked about the challenges they face and interventions or policies to reduce the effects of COVID-19.

The data collected were cleaned and analysed using STATA 16. The analysis covered descriptive statistics and gross margin (GM) to show the effect of the pandemic on livelihood outcomes, such as AIVs yield and incomes of smallholder farmers and traders. The income earned by farmers and traders was estimated using gross margin (GM) approach. The gross margin was calculated using the 2019 data and 2020 figures estimated using the change in production and sales. GM was calculated using the following formula:

1 $${\text{GM}}_{i} = {\text{TR}}_{i} - {\text{TVC}}_{i} ,$$

where $\mathrm{GM}$ is the gross margin; the difference between the total revenue and total variable cost; $\mathrm{TR}$ is the total revenue; the product of output price and quantity of output produced. $\mathrm{TVC}$ is the total variable cost; the difference between the total cost and total fixed cost. In computing the GM, such variables as total production, total area planted, cost of purchased inputs, quantity and value of sales were considered.

## Results and discussion

### Farmer and trader characteristics

About 41% of the sampled farmers were male while 59% were female. The average household size for the sampled households was 5, which is consistent with the Kenya Population and Housing Census of 2019. About 83% of the traders were women while 17% were men. These results indicate that AIV production and trade in Kenya is dominated by women, which could potentially mean that COVID-19 has disrupted their livelihoods more and widened the existing economic gender inequalities. Over 50% of the traders interviewed were from Nairobi which is a growing market and consumption hub for the AIVs. The rest of the traders were from Kiambu (18%), Migori (12%), Vihiga (12%) and Kisumu (9%).

Other household and trader characteristics are summarized in Table 3.

**Table 3:** Characteristics of farmers and traders of African indigenous vegetables

Characteristic	Farmers	Traders
Age (years)	51	51
Years of schooling	9	9
Experience (years)	17	9
Household land size (Ha.)	0.5	–
Credit access (% of farmers)	9	–
Number of observations (*N*)	244	246

On average, the AIV farmers were middle-aged (51 years), with only 9 years of schooling and a lot of experience in farming spanning 17 years. The average household landholding size was 0.5 hectares. Although credit is important for purchase of farm input and agricultural technologies, only 9% of the AIV farmers had access to credit, predominantly from informal savings and credit groups. The traders were 51 years old, on average, and had 9 years of formal schooling and business experience.

**Fig. 2:**
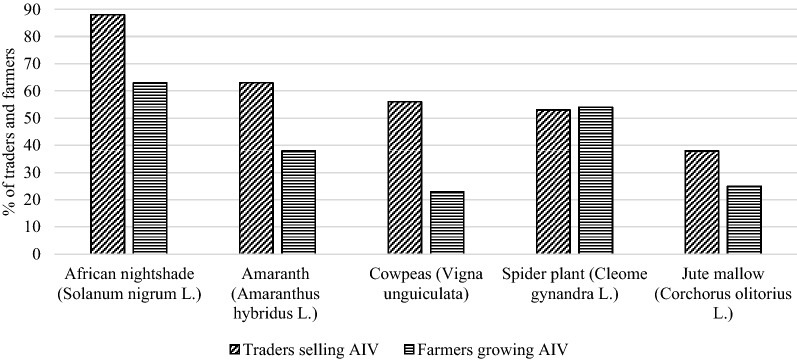
African indigenous vegetables grown and traded

The sampled farmers and traders grew and sold at least two of the five major AIVs in Kenya: African nightshade (*Solanum nigrum *L*.*)*, *Amaranth (*Amaranthus hybridus *L*.*), Cowpeas (*Vigna unguiculata *L*.*), Spider plant *(Cleome gynandra *L*.)*, Jute mallow (*Corchorus olitorius *L*.*) (see [4] for details). Figure 2 provides a summary of proportion of farmers and traders dealing in each of the AIV varieties.

Direct consumers constituted the largest proportion of the buyers. About 90% of the traders indicated that the largest proportion of their buyers were the direct consumers. About 38% of the traders also sold to other traders while only 8% sold to institutions such as schools and hospitals. This clearly indicates that the AIV value chain is fairly short, and that the vegetables are sold fresh with minimal value-addition, if any. Indeed, freshness was reported as the most important factor that buyers of AIVs consider. Other important considerations by the buyers were cleanliness of the vegetables were and leaf size (larger leaves being more preferable).

### Status of AIVs production and trade during COVID 19

During the pandemic 19% of the farmers did not grow any AIVs. Those who were growing AIVs in the current season had dedicated 0.35 acres on average to AIV production. While 25% of the farmers indicated that this was an increase in the area of land cropped with AIVs, 75% indicated it was a decrease (Table 4). Similarly, 18% of traders had suspended their trade due to COVID-19 pandemic. About 82% were, however, still running their businesses. Of the operating traders, 98% indicated that they had registered a decline in sales volume while 2% indicated that they had recorded an increase in sales volume. These findings are consistent to those reported in Ethiopia where traders in Addis Ababa reduced the volume due to the travel bans [7].

**Table 4:** Changes in farmers’ land area allocated to AIVs and trader’s sales volume due to COVID 19

	Farmers’ land		Traders’ sales volume	
	Proportion of farmers (%) reporting		Proportion of traders (%) reporting	
	Decrease	Increase	Decrease	Increase
Framers or traders reporting increase or decrease	75	25	98	2
Rate of increase or decrease				
Less than 25%	28	46	8	75
25–50%	40	46	30	25
50–75%	18	6	34	0
More than 75%	14	2	28	0
Number of observations (*N*)	148	50	197	4

Overall, among the farmers who had planted AIVs during the season, a reduction of 42% of land area cropped was registered. The farmers who recorded an increase in the land area cropped had an increase of about 28%. Treating the 46 farmers who did not plant AIVs at all as 100% reduction, the percentage drop of land area under AIVs becomes 56%. Thus, for the entire sample, the proportionate drop in the land area under AIVs is 39%.

Most farmers attributed the reduction of land area under AIVs to unavailability of seed (47%) and shortage of farm labour (50%) both of which are direct effects of the lockdown measures. Among the farmers who did not plant AIVs at all, 68% attributed it to excess rainfall and flooding in their farms while 32% cited unavailability of farm inputs such as seed and fertilizer, and shortage of funds to invest in farming. This is consistent with previous studies on pandemics and how they impact agricultural production (see [20–22, 42]). Key informants confirmed that access to farm inputs had become more difficult and more expensive with the emergence of COVID-19. Traders whose businesses remained open during the pandemic experienced reduced trade volumes of about 65% consistent with other studies [16, 43].

### Impact of COVID-19 on AIV production and profits from AIV trade

Smallholder farm households rely mainly on household/family labour and therefore, closure of schools and colleges potentially led to increased household labour supply. In our study, a paltry 3% of farmers had problems with farm labour although some studies in East Africa and China showed that the pandemic affected labour availability in agricultural sector and informal sector, e.g., small and medium-sized enterprises (SMEs), this include casual labourers supporting on-farm planting or harvesting activities (including migrant labourers), transport operators, petty traders, market vendors, and village-based loan and credit operators [36, 44, 45].

The greatest pathway through which COVID-19 hit the AIV farmers in Kenya is the markets. Most County governments closed the local open-air markets. At the same time, middlemen who purchase the produce at farm gate for urban markets were affected by reduced availability of public transport. The farmers interviewed reported that their produce got wasted due to lack of buyers, inability to value add or process and lack of improved storage facilities. Vegetable farmers in Ethiopia reported that during COVID 19 they had to leave some vegetables in the field to rot due to the lack of buyers [7]. About 36% of farmers reported shortage of farm inputs such as quality seeds and fertilizers. A survey in Ethiopia also indicated that farmers experienced shortage of inputs and increasing prices of inputs crucial for vegetable production such as fungicides, insecticides, herbicides, fertilizers, and improved seeds [7, 45, 46].

In addition, 73% of traders indicated that they had experienced a reduced number of AIV buyers. This is expected because many potential customers were working from home and convenience purchases declined. Usually AIVs are generally more expensive compared to other vegetables. Thus, in a situation of reduced incomes, demand for them would decline. Shortage of supply of AIVs affected 57% of the traders, and this must have been associated with increased cost and difficulty in transporting the vegetables from upcountry to, especially urban markets. Previously, most small-scale traders relied on public commuter buses for transportation of AIVs to urban markets. At the time of the survey, these buses were not in operation and traders had to rely on hired means of transport which was more expensive and uneconomical for small-scale traders. Reduced and/or controlled business opening due to the lockdown and curfew measures, affected 34% of the AIV traders.

Majority of AIV farmers and traders were affected by COVID-19, reducing the production and trade volumes (Table 5). Production of AIV declined by 39% while trade volumes and profits reduced by 65%. Farmers experienced shortage of quality seeds and fertilizer, leading to either reduced acreage and/or application of fertilizers, and use of recycled seeds which are likely to manifest in reduced quantity and quality of AIV outputs (Table 6). A study by [8] reported a decline in the vegetable production in India due to lack of inputs and harvest labour as a result of COVID-19 crisis. Similarly, limited access crucial inputs could have led to decline in farmers’ yield, a situation reported in India, Ethiopia and Mali [8, 45, 46]. A recent study in nine countries in Eastern and Southern Africa among bean farmers, aggregators, processors, bean regional co-ordinators, and mechanization dealers reveal that COVID-19 and government restrictions had impacted the availability and cost of farm inputs and labour, distribution, and consumption of beans [45]. Studies specifically on fruits and vegetable producers and vendors in Kenya revealed a widespread drop in yields and sales, respectively, with sales dropping by up to 70% [47]. The drops were mainly attributed to curfews and travel restrictions, which rendered critical farm inputs unavailable and prevented potential buyers from accessing cross county markets, as well as limiting the hours of business operation. These findings contribute to the growing evidence a cross several countries of the immediate and longer term impacts of COVID-19 on the food system with low- and middle-income countries suffering the most due to their weak health system and poor economic resources [48]. The impacts could be worse because only 26% of the farmers did nothing to mitigate the effects of COVID-19 on their production of AIVs.

**Table 5:** Impact of COVID-19 on AIV production and trade volumes and profits

	2019	2020	Difference	% impact
Farmers: study area^a^				
Total area planted on AIV (acres)	270	165	(105)	(39)
Production (kg)	3,15,646	1,92,544	(1,23,102)	
Farm profit/gross margin per acre	55,543	33,881	(21,662)	
Projections for the whole country				
Total area planted on AIV (acres)	5,85,338	3,57,056	(2,28,282)	(39)
Production in kg	9,96,567,547	6,07,906,203	(3,88,661,343)	
Traders: study area^a^				
Total quantity traded per day (kg)	50,909	17,818	(33,091)	(65)
Net profit/traders/day	1010	353	(656)	

^a^Kirinyaga, Migori, Vihiga, Nairobi, Kiambu and Kisumu. Data for calculating the effect of COVID-19 was obtained from FAO Statistics, 2020 and Tegemeo Agricultural Policy Research and Analysis Program (TAPRA, 2014) of Egerton University-a study done in 37 Counties in Kenya

**Table 6:** COVID-19 impact pathways on AIV farmers and traders

Farmers		Traders	
Pathway	Farmers affected (%)	Pathway	Traders affected (%)
Reduced access to farm labour	3	Reduced business opening	34
Reduced access to quality seeds	29	Reduced customers	73
Reduced access to produce market	87	Shortage of employees	1
Scarcity of fertilizers	17	Shortage of supply	57
Number of Observations	213	Number of observations	196

### Coping and mitigation strategies

Coping with the effects of COVID-19 was a major challenge for the traders. About 56% of the traders were unable to undertake any coping measures, 40% of them switched to other livelihood activities while 6% resorted to borrowing. Further results indicate that 58% of the farmers switched to other activities to improve their resilience while 30% resorted to family labour to bridge the farm labour gap and to cut down the cost of production. To cope with labour shortage, 9% of the traders reverted to use family labour rather than hired labour. In view of, farmers and traders were asked to propose measures to minimize the adverse impacts of COVID-19 pandemic (Table 7).

**Table 7:** Suggested ways to cushion AIV farmers and traders against adverse impacts of COVID-19

Farmers		Traders	
Measure	%	Measure	%
Government to provide seed and fertilizers	72	Regulated opening of the markets	96
Provide market for produce	56	–	–
Offer transport to market	25	–	–
Allow regulated opening of local markets	68	–	–
Suspend Cess on agricultural produce	5	Tax relief	11
Provide free PPE and sanitizers to traders	15	Provide free PPE	42
Number of observations	213		196

Nobody really knows when COVID-19 will be contained and therefore it is not easy to predict how long the businesses will take to bounce back. In the interim, however, 96% of the AIV traders thought that their businesses would benefit greatly if the government implemented some regulated reopening of the local fresh food markets. Another 42% of the traders suggested that the government could consider providing free personal protective equipment (PPE) and hand sanitizers to the traders to help them continue with their business. A few traders (11%) suggested that the traders could be cushioned from the effects of COVID-19 through tax relief. The traders are demanding this in response to the tax relief the government of Kenya provided to all Kenyan employees, employers and on goods and services. The government reduced maximum individual tax from 30 to 25%, resident corporation tax from 35 to 30%, value added tax from 16 to 14% among other measures [10].

Farmers recommended provision of quality seed and fertilizer as the most important intervention to boost production to meet the market demand for AIVs. Many farmers (68%) also thought that opening the local markets even in a regulated manner would be important in enabling them to sell the fresh vegetables, especially because movements to major urban markets had been curtailed, leading to a drop in traders buying AIVs at farm gate. An alternative intervention, suggested by 56% of the farmers, would be for the government to provide market through its own agencies or by facilitating private sector agencies. Other possible interventions, though suggested by fewer farmers, were provision of transport to the urban markets, provision of free PPE and hand sanitizers, and suspension of agricultural cess or tax.

## Conclusion

The results of this study showed that the COVID-19 pandemic and the measures for its containment affected the production and trade of African indigenous vegetables in Kenya. It led to disruption in supply of inputs, flow of products to the markets and actual trading. About 20% of farmers and 18% of trader stopped growing and trading in AIVs, respectively. Farmers that continued to grow AIVs reduced the land area under AIVs (75% of them) and increased the use of in-house family labour because hired labour became less available. Restrictions of markets operations led to reduction in number of buyers and reduction in traded volumes of AIVs. About 73% of traders cited reduced number of customers for their produce. To cope with the impacts of the pandemic 40% of the traders switched from selling in AIVs to other enterprises. About 56% of the traders indicated that had been unable to cope with the impacts of the pandemic in any specific way. Among the farmers, 58% indicate that they switched to other enterprises which 30% indicated that they resorted to using family labour to cope with reduced labour availability. These finding indicate that the pandemic affected the production and supply of an important source of nutrition among the Kenya population. Such an effect is not desirable in pursuit of the goal of food security and nutrition.

To ameliorate the impact of COVID-19 pandemic on production and supply of AIVs we recommend that national and county governments to take measures to cushion minimize the effect and enable the sector to recover. These measures should address both the short-term and long-term impacts to ensure the long-term recovery and growth of the AIV sub-sector. In the short-term, the respective County governments can put in place, and support implementation of public health safety protocols in the open-air markets. For instance, provision of sanitization facilities, social distancing protocols and other regulations required to make markets fully operational. The governments should consider suspending agriculture cess to reduce cost of transporting AIVs to the market. Other targeted support such as fertilizer subsidies currently focusing on maize could be extended to cover African indigenous vegetable producers during pandemic. Furthermore, we recommend that the on-going public vaccination programme by government to prioritize traders and producers by designating them in the priority social groups because of the critical role they play in ensuring access to nutritious food.

This study was conducted at the early stages of the onset of the pandemic in Kenya. Over the past 1 year, the pandemic has spread and several waves of increase and decrease experienced. It is not clear what the medium- and long-term effects on AIV production and trade has been. We recommend future studies to focus on long-term dynamics of the pandemic on agriculture value chain in the context of developing countries.

## Data Availability

The data used in this study are available on request.

## Abbreviations

AIV: African indigenous vegetables
BAU: Business as usual
EVD: Ebola virus disease
GDP: Gross domestic product
MERS: Middle East respiratory syndrome
MFI: Microfinance Institutions
NMK: National Museums of Kenya
NPI: Natural Products Industry Initiatives
SARS: Acute respiratory syndrome
SLF: Sustainable livelihoods framework
SME: Small and medium enterprises
